# Rotation in an Enantiospecific Self‐Assembled Array of Molecular Raffle Wheels

**DOI:** 10.1002/anie.202107708

**Published:** 2021-11-22

**Authors:** Dennis Meier, Abhishek K. Adak, Peter Knecht, Joachim Reichert, Sourav Mondal, Nithin Suryadevara, Senthil Kumar Kuppusamy, Keitaro Eguchi, Matthias K. Muntwiler, Francesco Allegretti, Mario Ruben, Johannes V. Barth, Shobhana Narasimhan, Anthoula C. Papageorgiou

**Affiliations:** ^1^ Physics Department E20 Technical University of Munich (TUM) James Franck Strasse 1 85748 Garching Germany; ^2^ Theoretical Sciences Unit & School of Advanced Materials Jawaharlal Nehru Centre for Advanced Scientific Research, Jakkur Bangalore 560054 India; ^3^ Institute of Nanotechnology Karlsruhe Institute of Technology (KIT) Hermann-von-Helmholtz-Platz 1 76344 Eggenstein-Leopoldshafen Germany; ^4^ Institute for Quantum Materials and Technologies Karlsruhe Institute of Technology (KIT) Hermann-von-Helmholtz-Platz 1 76344 Eggenstein-Leopoldshafen Germany; ^5^ Paul Scherrer Institut 5232 Villigen PSI Switzerland; ^6^ Centre Européen de Sciences Quantiques (CESQ) Institut de Science et d'Ingénierie Supramoléculaires (ISIS) 8 allée Gaspard Monge, BP 70028 67083 Strasbourg Cedex France

**Keywords:** dynamics, enantioselectivity, host–guest systems, monolayers, self-assembly

## Abstract

Tailored nano‐spaces can control enantioselective adsorption and molecular motion. We report on the spontaneous assembly of a dynamic system—a rigid kagome network with each pore occupied by a guest molecule—employing solely 2,6‐bis(1H‐pyrazol‐1‐yl)pyridine‐4‐carboxylic acid on Ag(111). The network cavity snugly hosts the chemically modified guest, bestows enantiomorphic adsorption and allows selective rotational motions. Temperature‐dependent scanning tunnelling microscopy studies revealed distinct anchoring orientations of the guest unit switching with a 0.95 eV thermal barrier. H‐bonding between the guest and the host transiently stabilises the rotating guest, as the flapper on a raffle wheel. Density functional theory investigations unravel the detailed molecular pirouette of the guest and how the energy landscape is determined by H‐bond formation and breakage. The origin of the guest's enantiodirected, dynamic anchoring lies in the specific interplay of the kagome network and the silver surface.

## Graphical abstract

A kagome network on the Ag(111) surface hosts guest molecules with enantiomeric selectivity. Each larger pore confines a single guest molecule. The punctuated pirouette of the guest molecule in this confined space is unravelled by a combination of density functional theory and variable temperature scanning tunnelling microscopy in real time at the single molecule level.

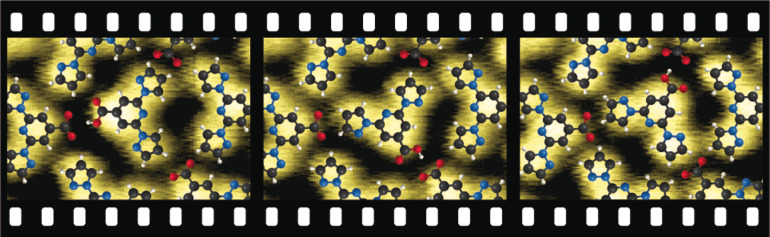

## Introduction

Inspired by biomolecular rotors that are omnipresent in nature, artificial molecular rotors have drawn attention in the field of nanoscience due to their potential application as functional molecular nanomachines. Mounting such devices on a surface in analogy to natural motors which operate at interfaces can expand their applicability. This has triggered intense scientific interest in the last decades.^[1]^ The pioneering work of Gimzewski and co‐workers demonstrated and established a methodological approach for the characterisation of such rotors by combining real space visualisation by scanning tunnelling microscopy (STM) with theoretical studies.^[2]^ It has been shown that the molecular motion on surfaces can be tuned by molecule–substrate interactions,^[3]^ as well as by supramolecular interactions.^[4]^ While isolated rotors have been the subject of intensive investigations, the programmed assembly and motion of molecular rotors is less explored, despite having advantages in applications such as novel sensors and signal processing.^[5]^ Different approaches have been shown to achieve this goal, including the use of bimetallic dislocation networks^[6]^ and molecular networks,^[7]^ to control the positioning of molecular rotors directly on the surface, as well as the two‐dimensional (2D) self‐assembly of molecular platforms, which were utilised for the arrangement of axially attached rotating units.^[8]^ Here we will demonstrate the regular assembly and motion of caged molecular rotors with enantiospecific stationary positions. An in‐depth analysis will reveal that the energy landscape of the rotation is influenced by a combination of hydrogen bonds to the host and site‐specific molecule–substrate interactions.

## Results and Discussion

**Figure 1:**
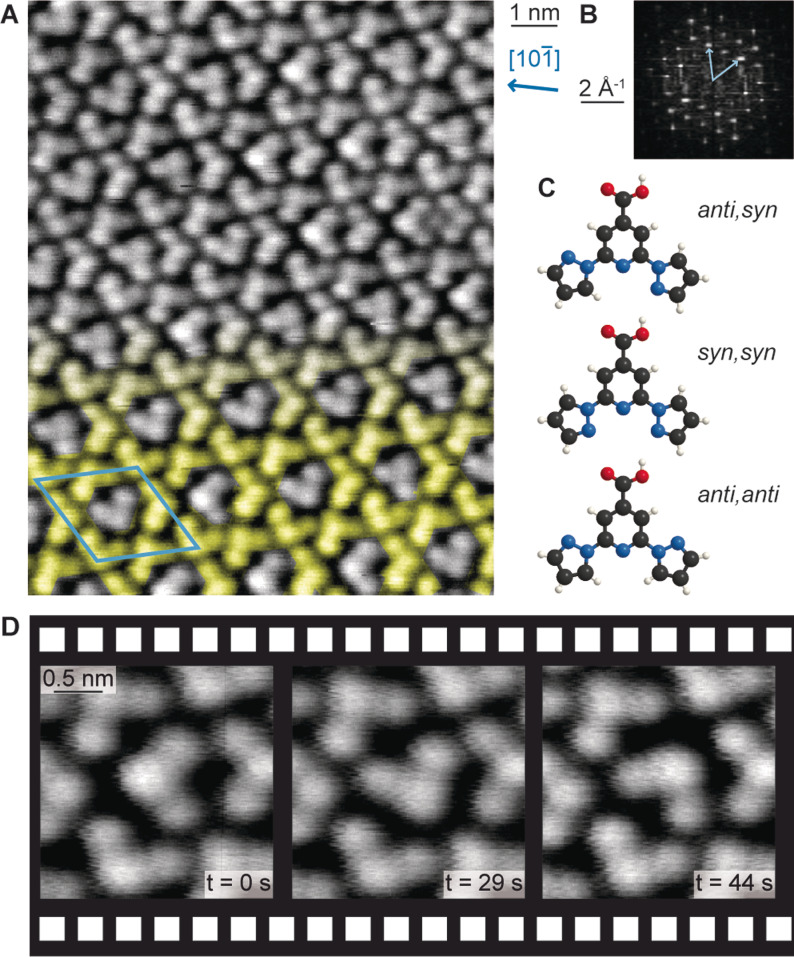
STM studies of the self‐assembled structure formed after annealing the bpp‐COOH layer on Ag(111) at 373 K and models of possible bpp‐COOH rotamers. A) Overview image (−625 mV; −40 pA; RT). The kagome network is highlighted in yellow on the bottom half of the image. The blue arrow indicates a Ag high symmetry axis in real space. B) Corresponding FFT. The light blue vectors identify the unit cell. The same unit cell is indicated on the real space data in (A). C) Ball‐and‐stick models of possible bpp‐COOH rotamers. C, N, O, H atoms are shown in black, blue, red and white, respectively. D) Consecutive images of the same molecules (−625 mV; −10 pA; RT) show different positions for the molecule in the larger kagome pore.

Our investigation was initiated by the discovery of an intriguing self‐assembly upon depositing bpp‐COOH (2,6‐bis(1*H*‐pyrazol‐1‐yl)pyridine‐4‐carboxylic acid) on Ag(111). The bpp‐COOH molecules were too mobile to image by STM investigations performed at 200 K and above, following deposition at room temperature (RT). However, annealing at 373 K stabilised a complex arrangement which could be imaged at RT (Figure 1 A). To identify the long‐range periodic features, we inspected the corresponding fast Fourier transform (FFT) showing distinct spots (Figure 1 B). These correspond to a unit cell which can be approximated by the epitaxial matrix of $\left(\begin{array}{cc} 8 & 2\\ -2 & 6 \end{array}\right)$ on the Ag(111) lattice, corresponding to a hexagonal overlayer twisted anticlockwise by 14° with respect to the substrate lattice.^[9]^ Correlating the reciprocal space periodicities to the real‐space data, we identify a motif containing four molecules. Three of these molecules are arranged regularly in a kagome pattern (Figure 1 A, highlighted in yellow). The fourth one appears in the pore of the kagome pattern. The orientation of the molecules in the pores is non‐periodic, causing local variations in the long‐range order.^[10]^ Consecutive images revealed alterations of the same guest molecule between different orientations (Figure 1 D and Supporting Information Movies S1). In contrast to a Brownian ratchet,^[11]^ no preferred directionality of the switching is expected for the thermally activated motion of the guest molecule in thermal equilibrium. An analysis of the rotational events showed no clear preferential direction (see Supporting Information Figure S1). Thus the supramolecular kagome is considered as a 2D matrix hosting single guest molecules in various orientations, reminiscent of the assemblies of a triangular discotic liquid crystal on graphite^[7a]^ and trimeric supramolecules of bisphenol A on Ag(111).^[7e]^

**Figure 2:**
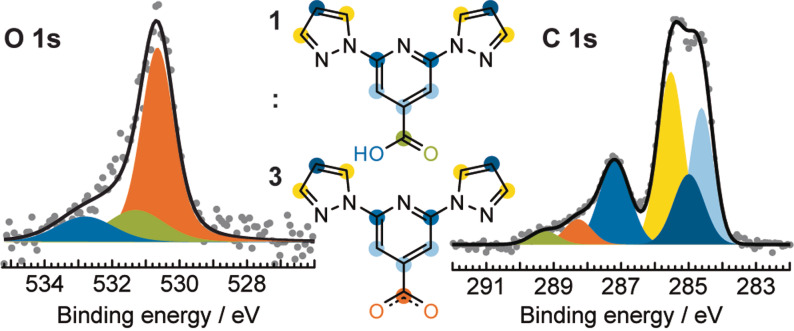
XPS characterisation of the host–guest architecture. Fitted O 1s (left) and C 1s (right) core‐level spectra corresponding to a single layer of bpp‐COOH on Ag(111) annealed at 373 K. The spectra are deconvoluted into components assigned to the chemically inequivalent atoms as marked by the colour indications in the chemical formulas (inset). A ≈6:1:1 ratio of intensities in the fitted O 1s region indicates the presence of three bpp‐COO^−^ moieties constituting the kagome (host) network and one bpp‐COOH moiety acting as a guest inside the pores of the host.

To identify the chemical state of the molecules within this self‐assembly, we carried out X‐ray photoelectron spectroscopy (XPS) measurements. The most informative core‐level region is the O 1s region (Figure 2, left). The signal is composed of three components in ratios of ≈6:1:1, in order of increasing binding energy. The higher binding energy components in a ratio of 1:1 are consistent with signals from the hydroxy and the carbonyl O atoms of the carboxylic acid moiety (bpp‐COOH), while the lower binding energy component can be assigned to O atoms in carboxylate moieties (bpp‐COO^−^).^[12]^ The C 1s signal (Figure 2, right) further supports the presence of carboxylate (orange) and carboxylic acid (green) moieties in a ratio of approximately 3:1 (detailed fitting parameters and respective assignment are summarised in Supporting Information Table S1). As the self‐assembled structure contains “stators” (arranged in a kagome pattern) and “rotors” in the same 3:1 ratio, we infer that the “stators” are chemically modified bpp‐COO^−^ molecules (bottom inset in Figure 2), and the “rotors” are pristine bpp‐COOH molecules (top inset in Figure 2). We therefore associate the stabilisation of the structure after annealing to 373 K with the formation of the carboxylate species, which is not present after the RT deposition (Supporting Information Figure S2).

**Figure 3:**
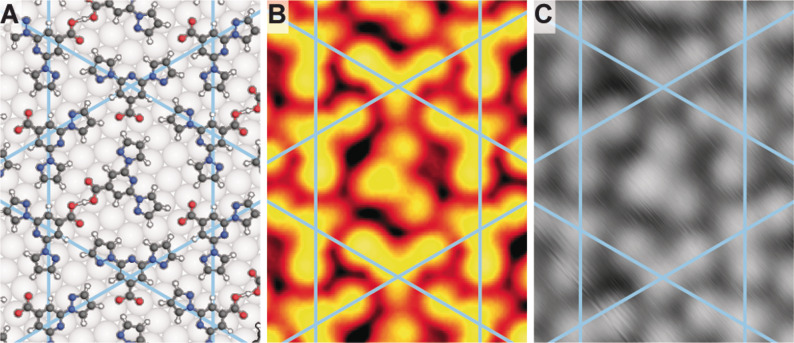
DFT‐optimised geometry and STM imaging of the host–guest network. The kagome network is indicated in all images by blue lines. A) Ball‐and‐stick model of the DFT‐optimised geometry. C, N, O, H, and Ag atoms are displayed in black, blue, red, white and grey, respectively. B) Simulated constant current STM image (−500 mV). C) Experimental STM image (−500 mV, 50 pA, 4 K).

After identifying the chemical state of the molecular components, we analysed their relative arrangement. From the STM contrast, we can identify the orientation of the pyridine–carboxy moiety (hereafter head group). However, the pyrazole orientation is not directly evident and similar contrast can arise from different planar rotamers (Figure 1 C and Supporting Information Figure S3). The highly symmetric nature of the kagome network suggests *syn*,*syn‐* or *anti*,*anti*‐configurations of the N atoms in adjacent pyridine–pyrazole units. As the *anti*–*anti* conformation is the most stable in the solid state,^[13]^ we do not anticipate the expression of a different rotamer after room temperature deposition. To ensure that this is also the preferred conformation on the surface following the deprotonation, we performed DFT modelling of the supramolecular network on Ag(111) with both configurations. The *anti*,*anti*‐structure is favoured by 1.86 eV per unit cell. A comparison of the hydrogen bonding schemes of the kagome *syn*,*syn*‐ and the *anti*,*anti*‐rotamers can rationalise a large energy difference between the two structures (see Supporting Information Figure S4). In addition, the preferred orientation of the guest molecule matches the STM images only for the *anti*,*anti*‐configuration (see larger pore of kagome pattern in Figure 3 and Supporting Information Figure S5 for DFT geometry optimisation of the *syn*,*syn*‐structure). The fcc hollow site was determined as energetically favoured for the pyridine of both *anti*,*anti*‐bpp‐COOH and bpp‐COO^−^ (Supporting Information Table S2) and was used to position the molecular units forming the kagome structure on Ag(111). Importantly, theoretically simulated STM images, obtained from DFT calculations, also reproduce the relative contrast difference of the head group compared to the pyrazole side groups. The pyridine–carboxylic acid appears brighter than the pyridine–carboxylate, reaffirming the assignment of chemically modified bpp‐COO^−^ tectons constituting the 2D kagome pattern on Ag(111) (Figure 3 B,C).

**Figure 4:**
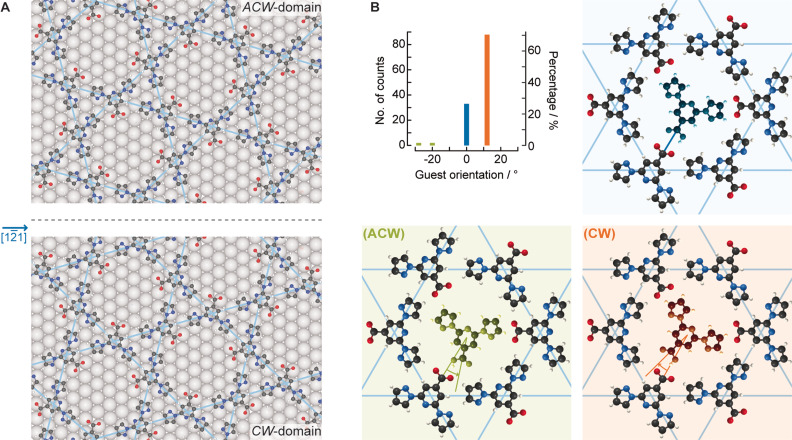
Chirality induction from the host to the guest. A) The host is chiral due to the twist angle between the kagome network and the Ag substrate. *ACW*‐domains (top) and *CW*‐domains (bottom) form as a result of the mirror symmetry along the Ag [1$\bar{2}$ 1] direction (dotted line). The host is represented with C, N, O, H and Ag atoms in black, blue, red, white and grey, respectively. B) Analysis of the clockwise rotation of guest molecules with respect to the nearest neighbouring host for head‐to‐head orientation. The counts represent the guest molecules of *ACW*‐domains at 4 K. The schematics illustrate different bpp‐COOH enantiomorphs of the guest molecules based on their detected geometry with respect to the kagome network. The guest molecules are coloured green (ACW), orange (CW) and blue (unidentified), to represent the different (ACW) and (CW) adsorbed bpp‐COOH enantiomorphs.

Subsequently, we analysed the orientation of the guest molecules in our “frozen” system (Figure 4 B) by inspection of STM images acquired at 4 K (see Supporting Information Figure S6 for an example). In general, this is found to be within 15° of the head‐to‐head orientation between the guest molecule and the host species. We expressed this as the angle of rotation of the guest molecule within the pore, with 0° being a guest carboxylic acid to host carboxylate head‐to‐head arrangement (indicated in the blue frame of Figure 4 B). Remarkably, we found a clear signature of an enantiospecific interaction for the guest molecules: 71 % were rotated clockwise, 26 % were not rotated and 3 % were rotated anticlockwise. The clockwise angle ((CW), orange in Figure 4 B) implies that the carboxylic H atom is expressed predominantly as a single surface enantiomer. It should be noted that bpp‐COOH is an achiral molecule, but confinement of the carbonyl carbon of bpp‐COOH to the surface plane gives rise to two surface enantiomers depending on the position of the carboxylic H atom. For the remaining molecules, the position of the carboxylic H atom of the bpp‐COOH guest cannot be deduced from the orientation (26 %, blue in Figure 4 B), whereas only 3 % exhibit clearly the anticlockwise bpp‐COOH enantiomer ((ACW), green in Figure 4 B). Therefore, the host network induces chiral organisation of the guest molecules. The origin of this effect can be found by looking at the registry of the kagome network on the substrate.

Enantiospecific molecular interactions on solid surfaces may stem from chiral steps or kinks^[14]^ or by molecular chiral modifiers.^[15]^ Unlike other reported 2D kagome networks,^[16]^ the one reported here is (if considering the unsupported layer) achiral, as is the bare Ag(111) surface. The origin of the enantiospecific interaction can be found in the twist when superposing the supramolecular structure to the substrate symmetry.^[17]^ Indeed, the superposition of two layers with a twist angle has been shown to give rise to chiral films^[18]^ with topologically protected chiral 1D edge states.^[19]^ Here, the rotated registry of the kagome network with respect to the substrate leads to *CW*‐ and *ACW*‐domains of the kagome network, which correspond to clockwise and anticlockwise rotations, respectively, of the kagome network with respect to the substrate lattice vectors (Figure 4 A). Note that the *CW*/*ACW* network is not to be confused with (CW)/(ACW) guest. Figure 4 B shows an analysis of solely *ACW*‐domains at 4 K. The *CW*‐ and *ACW*‐domains have opposite chiral induction effects on the guest molecule, resulting in different enantioselectivity (Supporting Information Figure S7). Importantly, the chiral induction is present even at RT, although the exact rotation angle of the guest has a broader distribution (Supporting Information Figure S8) between 0° and ±15°.

To verify the origin of this chiral induction, we investigated the effect of the Ag(111) substrate. DFT energy optimisation of the supramolecular structure in the absence of the Ag substrate shows that the (CW)‐ and (ACW)‐bpp‐COOH guest orientations are energetically degenerate, both have a minimum at 0°; and in addition, reflection planes at 0° and 60° transform the energy landscape of the (CW) into that of the (ACW). Similarly, on the bare achiral Ag(111) surface (with no kagome network present), the rotational energy landscapes of the (CW) and (ACW) have energetically degenerate minima; these however occur at different orientation angles for the two surface enantiomers and away from 0°. Accordingly, the reflection planes now occur at different angles. This means that the degeneracy of the minima of the (CW) and (ACW) enantiomers is broken when one adds the effect of both the substrate and the kagome network (Supporting Information Figure S9). Consequently, it can be inferred that it is the non‐zero twist angle between the kagome network and the Ag(111) which breaks the symmetry between (CW) and (ACW) and is thus responsible for the guest chiral induction.

The most frequently observed orientation of guest to host molecule matches well with the DFT‐optimised guest geometry. Having observed the switching of the position of the molecule in consecutive STM images at RT (Figure 1 A and Supporting Information Movies S1), we considered this a model system for a confined rotation controlled by H‐bonds. To better understand the dynamics of this system, we performed a dual experimental and theoretical investigation.

**Figure 5:**
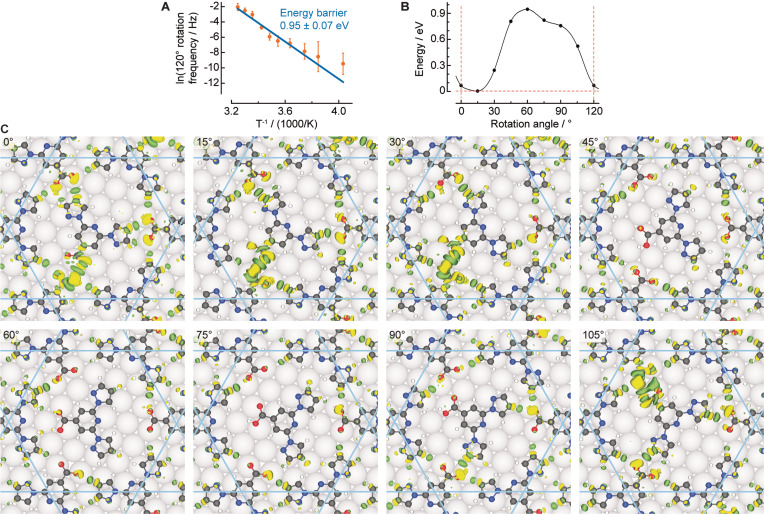
Dynamics of caged bpp‐COOH guest in the kagome network. A) Arrhenius plot of rotation frequency determined by STM data in order to deduce the energy barrier. B) DFT‐derived energy relative to the optimised geometry (guest at 15°) in the path leading to a guest rotation of 120° for (CW)‐guest in an *ACW*‐domain. The rotation angle represents the clockwise offset from the head‐to‐head orientation. C) The panels display the optimised geometries and superposed differential charge distribution at different rotation angles of the guest. (C, N, O, H, and Ag atoms are shown in black, blue, red, white and grey, respectively. Yellow and green lobes indicate electron accumulation and electron depletion, respectively, with isosurface values of 1.2×10^−3^ e bohr^−3^.)

We acquired systematically time‐resolved STM data of the switching events as a function of the temperature, tunnelling bias and tunnelling current. We find that the range of tunnelling conditions employed here does not influence the switching events, so we can use STM to follow the natural thermally activated rotation of the guest molecules within the nanopore (Supporting Information Figure S10). However, the possibility of tip‐induced activated rotation under different tunnelling conditions exists. No correlation of the rotations and positions of the neighbouring caged guests was noticed, we therefore considered them as independent rotors. We recorded the frequency of rotation events by ≈120° as a function of temperature (see plot in Figure 5 A and Methods in Supporting Information). With an Arrhenius equation fitting, the barrier is determined to be 0.95 eV ±0.07 eV with a corresponding pre‐exponential factor of ≈2.3×10^14±1^ s^−1^.

Our theoretical investigations provided a more detailed view of this process. In excellent agreement with the experimentally deduced value, a rotational barrier of 0.94 eV is computed (Figure 5). The main contribution to this barrier is the lateral bonding of the rotating guest to the kagome network pore (0.77 eV for rotation in the network alone, Supporting Information Figure S9). We identify different intermolecular H‐bonds in the charge redistribution maps corresponding to the charge density difference of the separate molecular constituents and the Ag substrate from the adsorbed host–guest system (Figure 5 C). The signature of an attractive interaction is a line of alternating yellow and green lobes, which represent isosurfaces of the same value of electron accumulation and depletion, respectively.^[20]^ The simulated 15° steps of a 360° rotation are animated in Supporting Information Movie S2. Five strong hydrogen bonds can be identified in the second most frequently observed geometry (Figure 5 C, direct head‐to‐head, 0°), where the kagome structure maximises its interaction with the guest molecule. At the global energy minimum, where the interaction of the guest molecule and the substrate is also optimised (0.07 eV lower in energy), three stronger and two weaker hydrogen bonds form, as illustrated by the charge redistribution visualised in Figure 5 C, 15°. As mentioned above, the twist angle between Ag(111) and the kagome network is responsible for this energy minimum. The energy maximum is found at a position where the guest does not have significant bonding with the host (Figure 5 C, 60°).

We also show that the charge transfer between the guest molecule and the host (comprising the kagome network and the Ag surface) correlates well with the height of the rotational barrier: the charge transfer is greatest at the minimum of the rotational energy landscape, and least for the most unfavourable guest orientation at 60° (Supporting Information Figure S11). Comparison with the confinement of an (ACW)‐bpp‐COOH guest in an *ACW*‐domain of the kagome network and its rotation (Supporting Information Figure S12) further supports the observation of chiral induction.

Interestingly, by scrutinising the optimised DFT geometries, the adaptability of the host with respect to the guest dynamic position in the optimised geometries becomes evident (Figure 5 B and Supporting Information Movie S2). Displacements of 0.78 Å can be detected for the centre of the pyridine ring, in line with dynamic porous features of responsive coordination polymers.^[21]^ As a footnote, we also noticed a statistically insignificant number (less than 1 %) of guests rotating too fast to be monitored with our scanning speed in our experimental data. We tentatively attribute these to potential bpp‐COO^−^ guests. Consideration of bpp‐COO^−^ guests by DFT found indeed a significantly reduced barrier of 0.11 eV for rotation of such guest molecules. However, the bpp‐COOH guest is significantly favoured by 0.81 eV/unit cell (Supporting Information Figure S13), further reinforcing our interpretation.

## Conclusion

We report on the spontaneous self‐assembly of bpp‐COO^−^ molecules on a well‐defined surface into a stable kagome network filled with bpp‐COOH guests in its pores. The registry of the kagome network on the Ag(111) substrate induces enantioselective organisation of the guest molecule inside the pores of the kagome network. The rotation of the guest molecule inside the pore is controlled by the kagome network: the chemical structure of the pore wall provides flappers that pin the guest molecule and prevent its unhindered rotation. The spontaneous formation of the host–guest assembly reported in this study is a model system for the investigation of delicate, dynamic, and enantiospecific interactions in confined spaces, which could help design enantioselective heterogeneous catalysts. We further anticipate that such ordered large‐scale networks with embedded individual switching units could be promising components for developing molecular nanodevices.

## Conflict of interest

The authors declare no conflict of interest.

## Supporting information

As a service to our authors and readers, this journal provides supporting information supplied by the authors. Such materials are peer reviewed and may be re‐organized for online delivery, but are not copy‐edited or typeset. Technical support issues arising from supporting information (other than missing files) should be addressed to the authors.

Supporting Information

Supporting Information

Supporting Information

Supporting Information
