# Supplementary material for: Rotation in an Enantiospecific Self‐Assembled Array of Molecular Raffle Wheels
Source: Angew Chem Int Ed Engl. 2021 Nov 22;60(52):26932–8. doi: 10.1002/anie.202107708 (PMC9299480; doi:10.1002/anie.202107708)
Supplement: Supplementary file 1 — Supporting Information [file ANIE-60-26932-s003.pdf]

Angewandte Chemie
Eine Zeitschrift der Gesellschaft Deutscher Chemiker

Supporting Information

Rotation in an Enantiospecific Self-Assembled Array of Molecular Raffle Wheels

Dennis Meier+, Abhishek K. Adak+, Peter Knecht+, Joachim Reichert, Sourav Mondal, Nithin Suryadevara, Senthil Kumar Kuppusamy, Keitaro Eguchi, Matthias K. Muntwiler, Francesco Allegretti, Mario Ruben, Johannes V. Barth, Shobhana Narasimhan,* and Anthoula C. Papageorgiou**

anie_202107708_sm_miscellaneous_information.pdf
anie_202107708_sm_Movie_S1_A.mp4
anie_202107708_sm_Movie_S1_B.mp4
anie_202107708_sm_Movie_S2.mp4

Contents

Methods . . . S-2
Description of Movies S1 . . . S-3
Description of Movie S2 . . . S-3
Table S1: O 1s and C 1s signals' deconvolution . . . S-4
Table S2: Adsorption energy . . . S-4
Figure S1: Counts of rotation angle of guest molecules . . . S-5
Figure S2: XPS data after deposition at room temperature . . . S-5
Figure S3: Plausible planar bpp-COO^- surface rotamers . . . S-6
Figure S4: Comparison of hydrogen bonding schemes of an *anti,anti* and a *syn,syn* kagome network . . . S-6
Figure S5: Simulation with *syn,syn* molecules . . . S-7
Figure S6: Exemplary determination of the orientation of the guest molecules . . . S-7
Figure S7: Chirality in host-guest network . . . S-8
Figure S8: Analysis of the orientation of guest molecules at room temperature . . . S-9
Figure S9: Energy landscapes for the rotation of guest molecules. . . . S-9
Figure S10: Rotation frequency as a function of tunnelling conditions . . . S-10
Figure S11: Charge transfer *vs.* rotation angle . . . S-11
Figure S12: Energy landscapes for the rotation of enantiomeric guest molecules in the *S*-domain of the kagome network . . . S-11
Figure S13: Rotation energetics of bpp-COO^- and bpp-COOH guest molecules . . . S-12

Methods

Synthesis of 2,6-bis(1H-pyrazol-1-yl)pyridine-4-carboxylic acid (bpp-COOH)

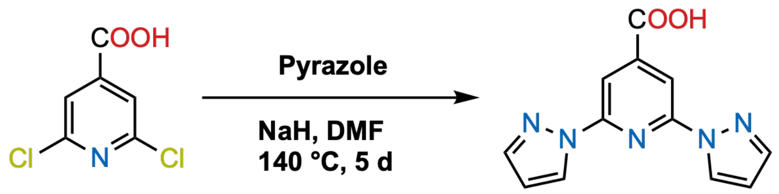

Freshly distilled DMF (200 mL) was taken in a three-necked round-bottomed flask and was degassed with argon for 1 h. Pyrazole (5.5 g, 80 mmol, 3 eq) was added and dissolved by stirring at room temperature. NaH (60 wt% in mineral oil, 6.5 g, 163 mmol, 6 eq) was added in 3 portions (Caution: evolution of H_2 !), which resulted in the formation of a white turbid solution and was stirred for 1 h at 100 °C. 2,6-dichloroisonicotinic acid (5 g, 26 mmol, 1 eq) was added in one portion and was stirred at 130 °C for five days under the argon atmosphere. The solvent was removed under reduced pressure, and distilled water (100 mL) was added. The mixture was acidified by the addition of concentrated HCl. The formed precipitate was filtered and dried in the oven.

Yield: 3.98 g (68.3%)
ESI-MS, found (calcd): 254.0695 $[M-H]^-$, (254.0673); 210.0805 $[M-COOH]^-$, (210.0774).
IR (KBr, cm^{-1}): 3148, 2924, 2854, 2563, 1727, 1619, 1575, 1526, 1471, 1465, 1446, 1402, 1307, 1233, 1211, 1141, 1104, 1045, 990, 972, 946, 900, 858, 786, 766, 746, 683, 646, 604, 504.
^{1}H NMR (500 MHz, Acetone-d6) δ/ppm: 8.90 (d, 2H), 8.39 (s, 2H), 7.86 (d, 2H), 6.63 (dd, 2H).
^{13}C NMR (126 MHz, Acetone-d6) δ/ppm: 164.5, 151.06, 144.24, 142.83, 127.67, 108.62, 108.46.

Sample preparation

Samples were prepared *in situ* under ultra-high vacuum conditions. Briefly, the Ag(111) crystal was cleaned by multiple cycles of Ar^+ sputtering (1 kV, sample current of 8 μA, 20 min or 1 kV, sample current of 30 μA, 5 min) and subsequent annealing (710 K, 5 min). The molecules were outgassed in vacuum and then deposited via organic molecular beam epitaxy from a quartz crucible at 170 °C. The sample was kept at 300 K during evaporation and heated subsequently to 373 K.

Scanning tunnelling microscopy (STM)

STM measurements were performed with electrochemically etched tungsten tips. The tunnelling biases reported represent the sample bias.

For the measurements in the temperature range of 250 to 310 K, we utilised an Aarhus-type VT-STM housed in a home made UHV system (base pressure 2×10^{-10} mbar) in the Technical University of Munich. The data points for the Arrhenius plot (Figure 5a) are based on at least 10 consecutive STM images (7×7 nm^2, approximately 25 independent rotors per image). The acquisition time of each image was 10 s. The total number of observed rotation events was divided by the total number of rotors and the acquisition time in order to obtain an average rotation frequency

For the measurements at 4 K, the Omicron LT-STM (base pressure 1×10^{-10} mbar) adjoined to the SLS beamline was used. The histogram of the orientation of the guests molecules in Figure 4 has a bin width of 3°.

X-ray photoelectron spetrocroscopy (XPS)

Spectra were recorded with a Scienta EW4000 hemispherical electron analyser at 300 K at the PEARL beamline of the Swiss Light Source (SLS). We have used excitation energies of 400 eV for C 1s spectra and 640 eV for O 1s spectra. Measurements were performed in normal emission geometry and binding energy scales were calibrated by the Ag $3d_{5/2}$ line at 368.2 eV for O 1s spectra and by the Fermi edge for C 1s spectra.

Density functional theory (DFT) calculations

The calculations were performed using DFT as implemented in the Quantum ESPRESSO package [1]. A plane wave basis set was used, with cut-offs of 40 Ry and 400 Ry for wavefunctions and charge densities, respectively [2]. Interactions between ionic cores and valence electrons were described using ultrasoft pseudopotentials [3] . Exchange-correlation interactions were treated using the Perdew-Burke-Ernzerhof (PBE) form of the Generalized Gradient Approximation [4]. Dispersion (van der Waals) interactions were incorporated using Grimme's DFT-D2 method [5].

The Ag(111) support was treated using a three layer slab. A vacuum spacing of $\sim$ 15 Å was introduced in the z-direction (perpendicular to the surface). Brillouin zone sampling was restricted to the zone-center Γ for the large unit cells comprising the molecules adsorbed on Ag(111). Convergence was aided by making use of the Marzari-Vanderbilt smearing technique, with a width of 0.005 Ry [6]. Except for the constrained-minimization calculations performed to calculate the rotation barrier, all atomic coordinates, except for those in the bottommost Ag layer, were allowed to relax using the BFGS algorithm [7, 8, 9, 10]. The force convergence threshold was 0.03 eV/Å [11]. Charge transfer between guest molecules and the kagome network was calculated using the Bader charge-partitioning method [12, 13].

Description of Movies S1

Videos of consecutive STM images showing the motion of bpp-COOH molecules in the kagome structure at room temperature. (A) A short movie (42×42 Å^2, -0.62 V, 20 pA) depicting the guest molecules' mobility within the pores in grey and highlighting the kagome structure in yellow. The speed of the video is accelerated by a factor of 30 with respect to the real time sampling. (B) A longer movie (38×53 Å^2, 1.485 V, 100 pA). The speed of the video is accelerated by a factor of 50 with respect to the real time sampling.

Description of Movie S2

Video of the simulated 15° steps of a 360° rotation. C, N, O, H, and Ag atoms in black, blue, red, white and grey, respectively. Yellow and green lobes indicate electron accumulation and electron depletion, respectively.

Table S1: Deconvolution of O 1s and C 1s signals corresponding to submonolayer bpp-COOH on Ag(111) after annealing to 370 K. For comparison to the peak intensity (central column) the corresponding atomic percentage is displayed on the right column.

Peak	Energy / eV	Peak intensity / %	Atom percentage in a mixture of 3 bpp-COO^- : 1 bpp-COOH
O 1s: hydroxyl	532.8	13	12
O 1s: carbonyl	531.3	18	12
O 1s: carboxylate	530.7	68	75
C 1s: carboxyl	289.3	3	2
C 1s: carboxylate	288.3	5	6
C 1s: N – C – N (pyridine)	287.2	19	17
C 1s: C – N (pyrazol)	285.5	33	33
C 1s: pyrazol C	285.0	16	17
C 1s: pyridine C	284.6	24	25

Table S2: Adsorption energy of the *anti,anti*-rotamer at different adsorption sites as calculated by DFT.

Adsorption site (pyridine)	Isolated molecule	Isolated deprotonated molecule
FCC hollow	0.0 eV	0.0 eV
HCP hollow	0.03 eV	0.036 eV
Atop	0.036 eV	0.028 eV
Bridge	0.059 eV	0.057 eV

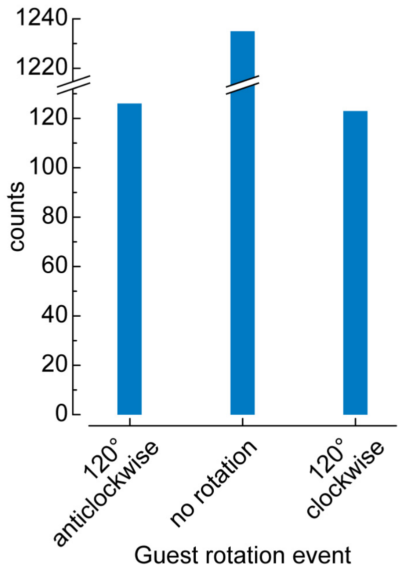

Figure S1: Counts of approximate guest molecule rotation events in consecutive STM images at room temperature. The data were taken in a kagome network domain twisted clockwise by 14° with respect to the substrate lattice. The acquisition time of the consecutive STM images was 15 s.

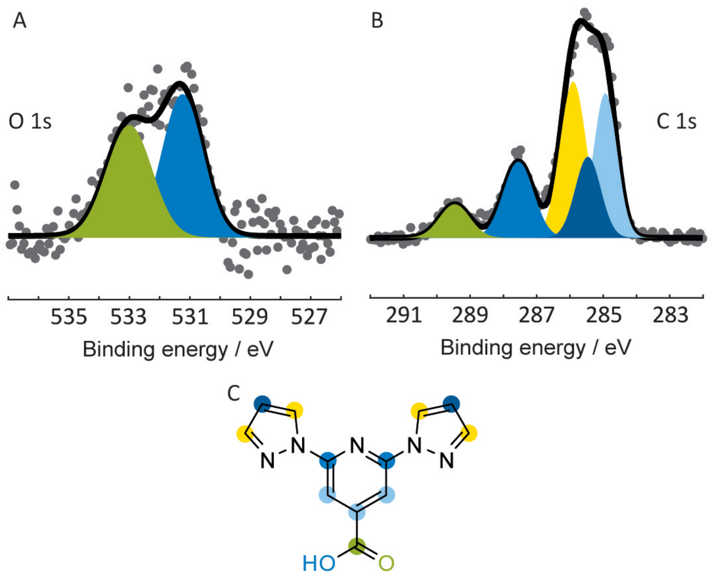

Figure S2: Fitted O 1s (A) and C 1s (B) core-level spectra corresponding to a single layer of bpp-COOH on Ag(111) after deposition at room temperature. The spectra are deconvoluted into components assigned to the chemically inequivalent atoms as marked by the colour indications in the chemical formula (C).

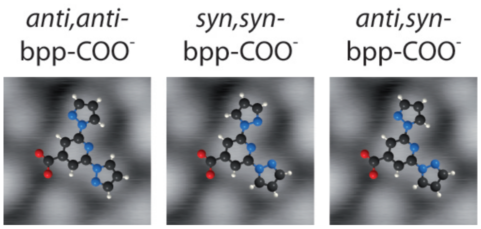

Figure S3: Plausible planar bpp-COO^- surface rotamers, indistinguishable by STM imaging. C, N, O and H atoms are shown in black, blue, red and white, respectively.

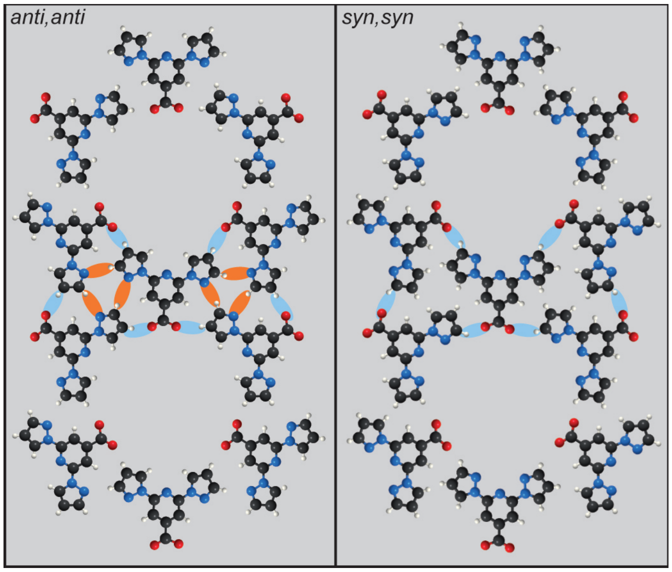

Figure S4: Comparison of hydrogen bonding schemes of an *anti,anti* and a *syn,syn* kagome network. The blue ellipses show hydrogen bondings. In the *anti,anti* kagome network the orange ellipses show possible additional hydrogen bondings. C, N, O and H atoms are shown in black, blue, red and white, respectively.

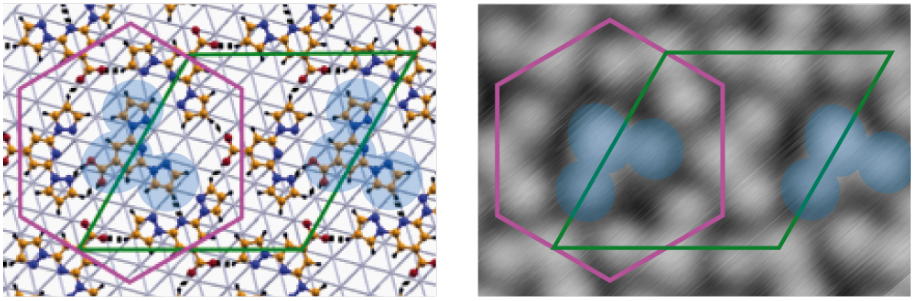

Figure S5: The *syn,syn*-host(bpp-COO$^-$)-guest(bpp-COOH) network on Ag(111). Relaxed structure from DFT (left), compared to STM data (right, -500 mV, 50 pA, 5 K). The green and magenta lines indicate the boundaries of a rhombus-shaped and hexagonal unit cell, respectively. The guest molecules are highlighted in blue. Black dotted lines indicate hydrogen bonds. Colour code for atomic spheres: C: yellow, N: blue, O: red, H: black. Grey lines indicate Ag-Ag nearest neighbour bonds in the topmost Ag layer.

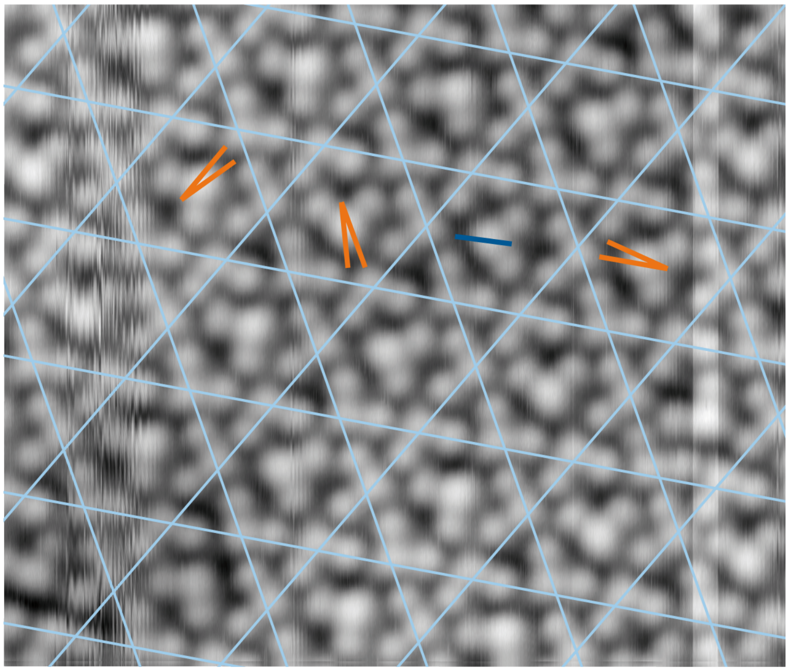

Figure S6: Exemplary determination of the orientation of the guest molecules (-500 mV, 50 pA) at 4 K. The data were taken in a kagome network domain twisted anticlockwise by 14° with respect to the substrate lattice. The dark blue line indicates a head-to-head orientation of the guest and the host. The orange lines indicate a +15° angle of clockwise rotation of the guest to the host molecule. The kagome network is indicated by light blue lines.

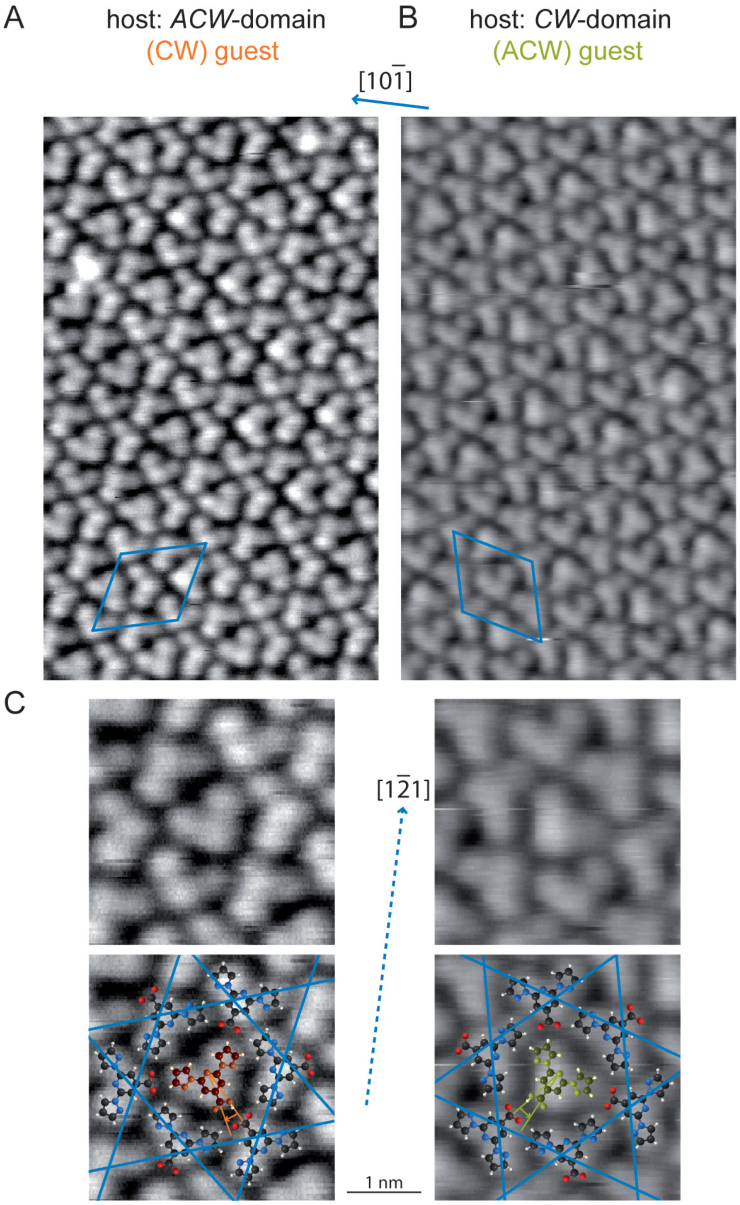

Figure S7: The two chiral kagome domains of the host-guest network. STM images (A, B: overview images, C: magnified images) of the anticlockwise (*ACW*) (-625 mV, 40 pA) and clockwise (*CW*) (1500 mV, 100 pA) kagome domains imaged at room temperature. The $[10\bar{1}]$ and $[1\bar{2}1]$ directions of the Ag(111) are indicated. The guest molecule is coloured differently, depending on its surface chirality. The *CW*- and *ACW*-domains result in enantiomeric separation of the guest molecules. Approximately 70% of the guest molecules exhibit the depicted enantiomer, as deduced by the relative rotation of host to guest. For around 30%, the surface enantiomer cannot be deduced.

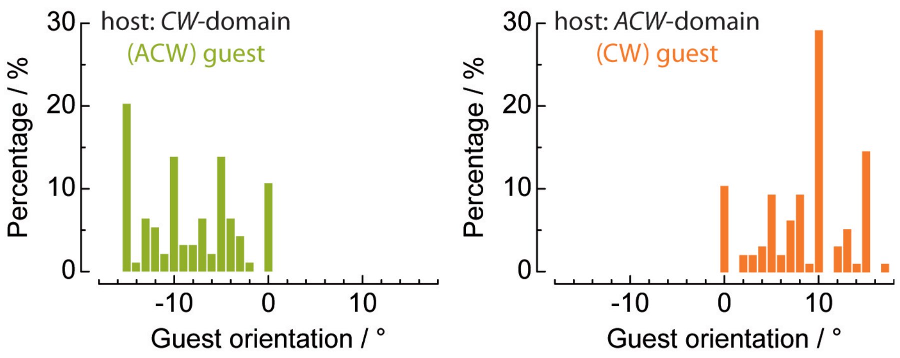

Figure S8: Analysis of the orientation of guest molecules with respect to the nearest neighbouring host for head-to-head interaction. The counts represent the guest molecules of CW-domains (left) and ACW-domains (right) at room temperature.

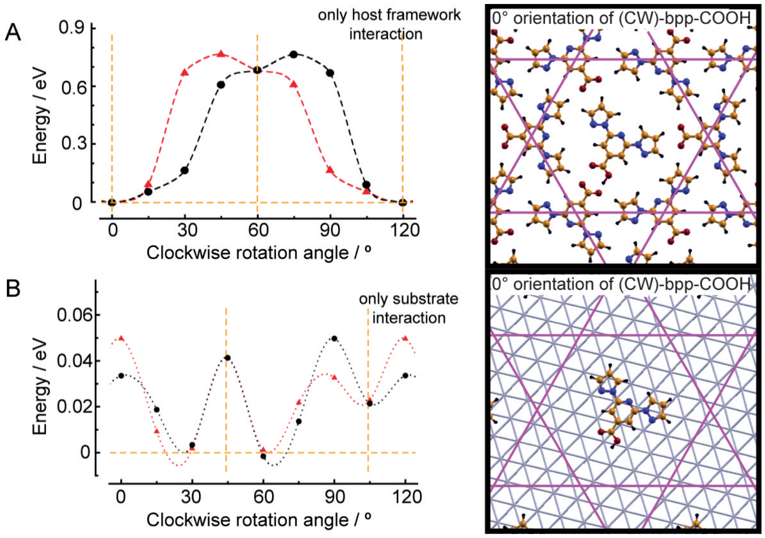

Figure S9: Energy landscapes for the rotation of guest molecules obtained by DFT calculations on an ACW-domain. (A) The rotation energy of the (CW)-(black) and (ACW)-(red) guest in the kagome network without the presence of the Ag(111). The rotational energy landscapes of the two enantiomers are energetically degenerate, being related by reflection planes at 0° and 60°, and with a minimum at a rotation angle of 0° for both enantiomers. (B) The rotation energy landscape of the (CW)-(black) and (ACW)-(red) guest on Ag(111) in the absence of the kagome network. The rotation energy of the two enantiomers has minima and reflection planes (indicated by dashed vertical lines) at different rotation angles from that in (A). Note that on adding the curves in (A) and (B) the degeneracy between the two enantiomers will be broken.

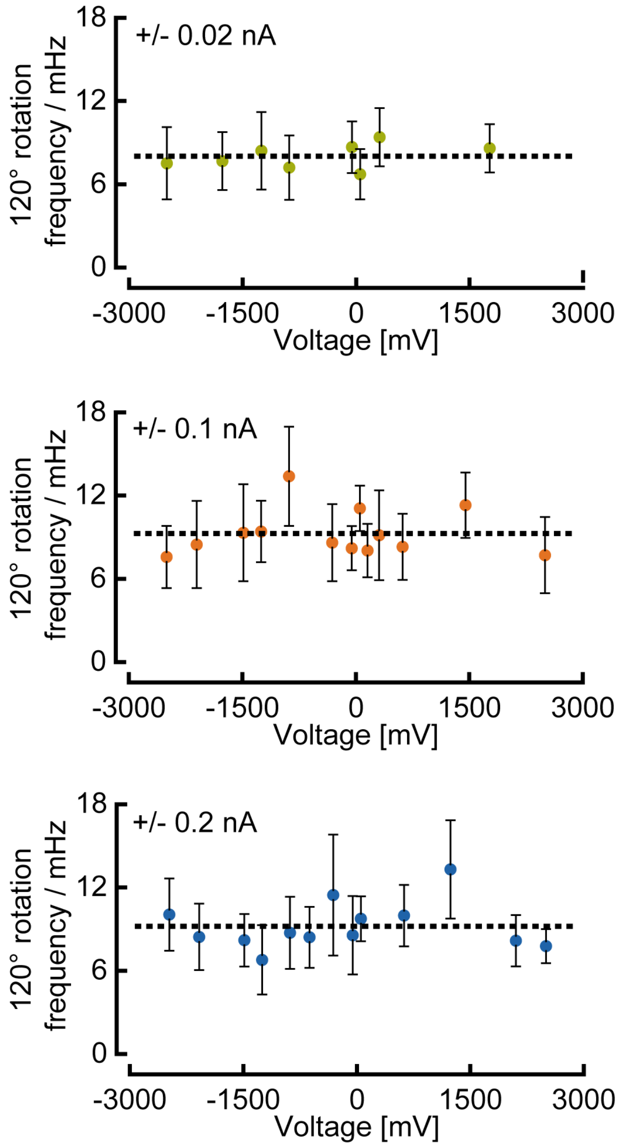

Figure S10: The detected rotation frequency showed no dependence in the tunnelling current range of 0.02 to 0.20 nA and in the tunnelling bias range of -3000 to 3000 mV.

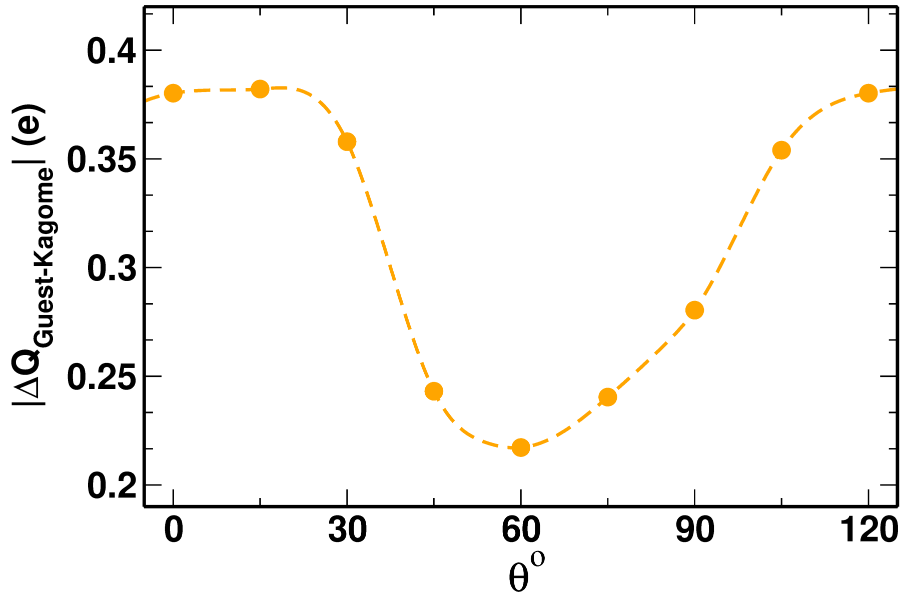

Figure S11: Charge transfer between the guest molecules and the kagome network *vs.* the guest rotation angle, θ. Note the correlation of the minimum of the rotation energy to the maximum of the charge transfer.

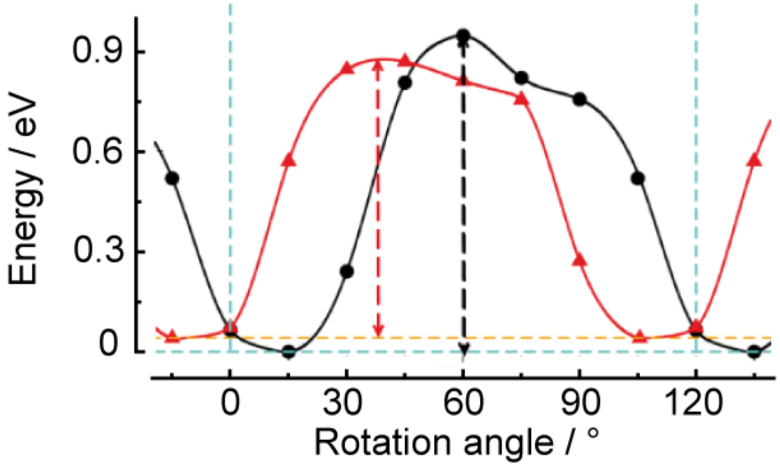

Figure S12: Energy landscapes computed from DFT for the rotation of (CW)-(black) and (ACW)-(red) guest in an *ACW*-domain of the kagome network on Ag(111). Note that the minimum for (CW)-guest is lower in energy than the minimum for (ACW)-guest.

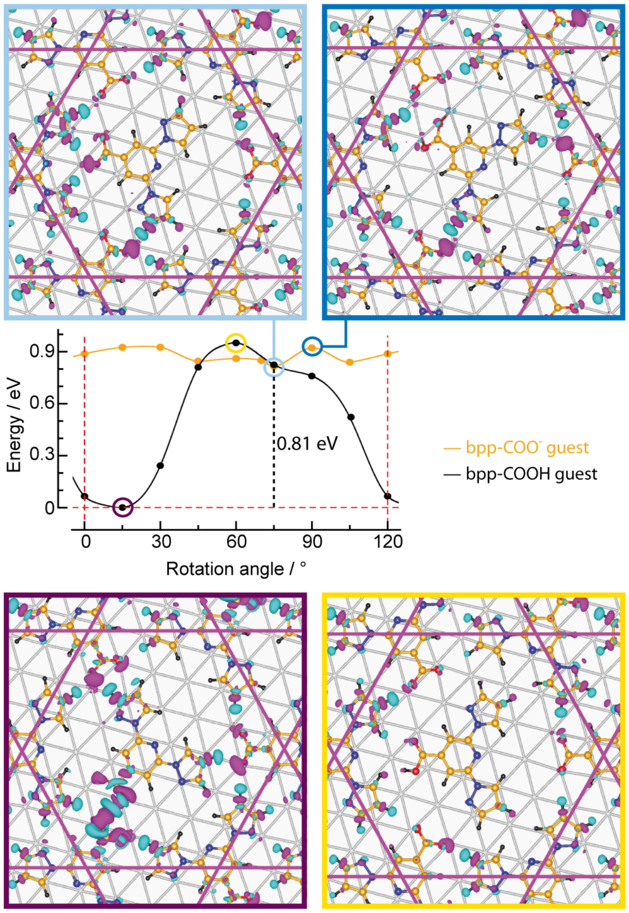

Figure S13: Gibbs free energy comparison of rotational barrier of guest molecules for deprotonated (yellow) and protonated (black) guest. For protonated (black) guest molecules, the Gibbs free energy $G = E_{tot}$, where E_{tot} is the total energy computed from DFT. For deprotonated (yellow) guest molecules, $G = E_{tot} + \frac{1}{2}\mu_H$ where μ_H is the chemical potential of hydrogen. Throughout the rotation, the protonated guest system is energetically favoured, except in the window between $50° - 75°$. The atomistic models show the DFT optimised geometries at the indicated guest orientations. Colour code for atomic spheres: C: yellow, N: blue, O: red, H: black. Grey lines indicate Ag-Ag nearest neighbour bonds in the topmost Ag layer. Each line of alternating magenta and cyan lobes indicates the formation of a hydrogen bond. The purple lines indicate the kagome lattice of the host molecules.

References

[1] P. Giannozzi, S. Baroni, N. Bonini,M. Calandra, R. Car, C. Cavazzoni, D. Ceresoli, G. L. Chiarotti, M. Cococcioni, I. Dabo, A. D. Corso, S. de Gironcoli, S. Fabris, G. Fratesi, R. Gebauer, U. Gerstmann, C. Gougoussis, A. Kokalj, M. Lazzeri, L. Martin-Samos, N. Marzari, F. Mauri, R. Mazzarello, S. Paolini, A. Pasquarello, L. Paulatto, C. Sbraccia, S. Scandolo, G. Sclauzero, A. P. Seitsonen, A. Smogunov, P. Umari, R. M. Wentzcovitch, *J. Phys.: Condens. Matter* **2009**, *21,* 395502.

[2] W. Kohn, L. J. Sham, *Phys. Rev.* **1965**, *140,* A1133–A1138.

[3] D. Vanderbilt, *Phys. Rev. B* **1990**, *41,* 7892–7895.

[4] J. P. Perdew, K. Burke, M. Ernzerhof, *Phys. Rev. Lett.* **1996**, *77,* 3865–3868.

[5] S. Grimme, *J. Comput. Chem.* **2006**, *27,* 1787–1799.

[6] N. Marzari, D. Vanderbilt, A. De Vita, M. C. Payne, *Phys. Rev. Lett.* **1999**, *82,* 3296–3299.

[7] C. G. Broyden, *IMA J. Appl. Math.* **1970**, *6,* 76–90.

[8] R. Fletcher, *Comput. J.* **1970**, *13,* 317–322.

[9] D. Goldfarb, *Math. Comput.* **1970**, *24,* 23–26.

[10] D. F. Shanno, *Math. Comput.* **1970**, *24,* 647–656.

[11] R. P. Feynman, *Phys. Rev.* **1939**, *56,* 340–343.

[12] G. Henkelman, A. Arnaldsson, H. Jónsson, *J. Comput. Mater. Sci.* **2006**, *36,* 354–360.

[13] R. F. W. Bader, Atoms in Molecules: A Quantum Theory, *Oxford University Press, Oxford,* **1990**.
